# Silencing homeobox C6 inhibits colorectal cancer cell proliferation

**DOI:** 10.18632/oncotarget.8703

**Published:** 2016-04-12

**Authors:** Meiling Ji, Qingyang Feng, Guodong He, Liangliang Yang, Wentao Tang, Xinyuan Lao, Dexiang Zhu, Qi Lin, Pingping Xu, Ye Wei, Jianmin Xu

**Affiliations:** ^1^ Department of General Surgery, Zhongshan Hospital, Fudan University, Shanghai, China

**Keywords:** colorectal cancer, proliferation, HOXC6, autophagy, mTOR

## Abstract

Homeobox C6 (HOXC6), a member of the homeobox family that encodes highly conserved transcription factors, plays a vital role in various carcinomas. In this study, we used a tissue microarray (TMA) consisting of 462 CRC samples to demonstrate that HOXC6 is more abundantly expressed in colorectal cancer (CRC) tissues than adjacent normal mucosa. Clinicopathological data indicated that higher HOXC6 expression correlated with poor overall survival and was associated with primary tumor location in the right colon, primary tumor (pT) stage 3/4 and primary node (pN) stage 1/2. Multivariate analysis showed that high HOXC6 expression was an independent risk factor for poor CRC patient prognosis. HOXC6 downregulation via lentivirus-mediated expression of HOXC6-targeting shRNA reduced HCT116 cell viability and colony formation *in vitro*, and reduced growth of subcutaneous xenografts in nude mouse. HOXC6 thus appears to promote CRC cell proliferation and tumorigenesis through autophagy inhibition and mTOR pathway activation.

## INTRODUCTION

A comprehensive understanding of the oncogenic mechanisms associated with colorectal carcinogenesis is critical for the development of innovative therapeutic strategies to treat this disease. Colorectal cancer (CRC) is the third most commonly diagnosed cancer in males and the second in females, with an estimated 1.4 million cases and 693,900 deaths in 2012 [1]. The specific genes and molecular mechanisms triggering CRC remain largely unknown.

Human homeobox (HOX) genes belong to the homeoprotein family of transcription factors, which play critical roles in embryonic development, cell morphogenesis and differentiation [2, 3]. Humans harbor 39 different HOX genes from four groups (HOXA, HOXB, HOXC, and HOXD). Deregulated HOX gene expression has been reported in many cancers, including lung, breast and ovarian tumors, sarcoma and leukemia. Many HOX genes are involved in tumor cell proliferation, metastasis and angiogenesis, and levels of these genes are correlated with patient outcome [4]. Thus, HOX genes are emerging as key players in a variety of cancers and are potential markers for disease diagnosis and targets for novel therapies. However, the specific mechanisms by which HOX genes contribute to the tumorigenic phenotype are incompletely described.

Homeobox C6 (HOXC6) is reportedly overexpressed in breast [5, 6], lung [7] and prostate tumors [8], along with leukemia [9], osteosarcomas [10] and medulloblastomas [11]. HOXC6 is a candidate gene for early prostate cancer diagnosis [12], and upregulated HOXC6 has been associated with poor survival in gastric cancer patients [13]. Inprostate cancer cells, HOXC6 knockdown reduced proliferation by inducing apoptosis [8]. HOXC6 activated Ap-1 binding to JunD and promoted cell proliferation in pancreatic cancer cells [14]. HOXC6 is also deregulated in human head and neck squamous cell carcinoma and modulates Bcl-2 expression [15]. In addition to its functions in cell proliferation and survival, HOXC6 regulated MDR-1 transcription to govern multidrug resistance [16]. Although HOXC6 is differentially expressed in metastatic CRC [17], its expression profile, function and relationship to prognosis in CRC are largely unknown.

Autophagy, a process of self-digestion in mammalian cells, occurs at low basal levels for the purpose of homeostatic functions such as protein and organelle turnover. However, autophagy is rapidly upregulated when cells need to generate intracellular nutrients and energy, such as during starvation, growth factor withdrawal or times of high bioenergetic demand [18, 19]. The target of rapamycin, TOR kinase, is a key autophagy regulator and the major inhibitory signal that shuts off autophagy in the presence of growth factors and abundant nutrients. Downstream of TOR kinase, a series of autophagy-related genes (ATG genes) are essential for the execution of autophagy [20]. Recent discoveries have pointed to autophagy deregulation as a novel feature central to the pathogenesis of human malignancy, and the role of autophagy in cancer is both dynamic and highly complex [21].

In the present study, we found that HOXC6 was highly expressed in colorectal cancer compared to adjacent normal mucosa. Clinicopathologic data indicated that higher HOXC6 expression correlated with poor overall survival (OS) and was associated with primary tumor location in the right colon, primary tumor (pT) stage 3/4 and primary node (pN) stage 1/2. In addition, HOXC6 knockdown prevented cell proliferation and simultaneously inhibited autophagy. We conclude that HOXC6 is a CRC oncogene that likely promotes tumorigenesis through autophagy.

## RESULTS

### HOXC6 was highly expressed in colorectal cancer tumor tissues

**Figure 1:**
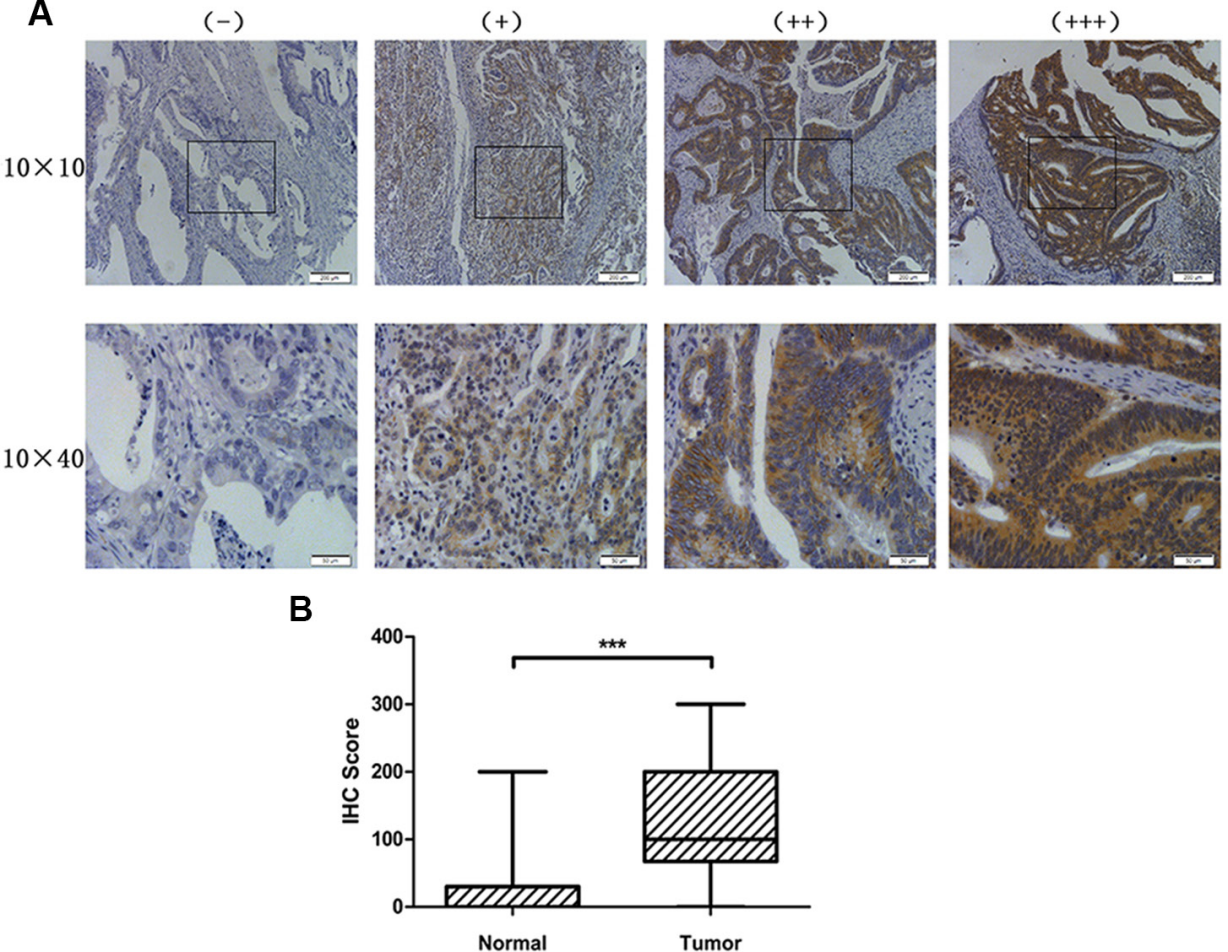
HOXC6 was highly expressed in CRC tumor tissues Representative images of HOXC6 IHC staining intensity: (−) no staining; (+) weak staining; (++) moderate staining; (+++) strong staining (**A**) HOXC6 IHC staining score was significantly higher in tumor tissues than in normal tissues (**B**) ***P < 0.001 via Wilcoxon signed rank test (matched pairs).

Primary tumor tissue samples from 462 patients were used in this study. HOXC6 protein expression was detected by immunohistochemistry (IHC) in a tissue microarray (TMA). Representative images (Figure 1A) show four different typical HOXC6 expression patterns. HOXC6 IHC scores in tumors were significantly higher than those in normal tissues (*P* < 0.001) (Figure 1B), suggesting that HOXC6 accumulated in CRC tissues.

### HOXC6 expression was associated with clinicopathological characteristics

For IHC score, the median value was defined as the cut-off between the HOXC6 high- and low-expression subgroups. A total of 238 samples exhibited high expression, and 224 exhibited low expression. High HOXC6 expression was associated with primary tumor location in the right colon (*P* = 0.036), primary pT stage 3/4 (*P* < 0.001) and primary pN stage 1/2 (*P* < 0.001). Patients with high HOXC6 expression more frequently received postoperative adjuvant chemotherapy (*P* = 0.055) because of higher tumor stage (Table 1).

**Table 1:** Relationships between HOXC6 expression in tumor tissues and patient/tumor clinicopathologic characteristics

	High (%) *n* = 238	Low (%) *n* = 224	Correlation coefficient	*P* value
Sex			0.020	0.664
Male	143 (60.1)	139 (61.2)		
Female	95 (39.9)	85 (37.9)		
Age - year			0.022	0.631
≤ 60	119 (50.0)	117 (52.2)		
> 60	119 (50.0)	107 (47.8)		
Preoperative CEA - ng/ml			0.053	0.254
< 5	117 (49.2)	122 (54.5)		
≥ 5	121 (50.8)	102 (45.5)		
Preoperative neoadjuvant treatment			0.019	0.677
No	171 (71.8)	157 (70.1)		
Yes	67 (28.2)	67 (29.9)		
Primary tumor site			0.120	0.036*
Right-sided	77(32.4)	51 (22.8)		
Left-sided	58 (24.4)	73 (32.6)		
Rectum	103 (43.3)	100 (44.6)		
Primary tumor size - cm			0.041	0.384
≤ 4	135 (56.7)	136 (60.7)		
> 4	103 (43.3)	88 (39.3)		
Primary histological type			0.056	0.227
Non-mucinous	200 (84.0)	197 (87.9)		
Mucinous	38 (16.0)	27 (12.1)		
Primary differentiation			0.052	0.259
Well to moderate	164 (68.9)	165 (73.7)		
Poor	74 (31.1)	59 (26.3)		
Primary pT stage			0.164	< 0.001*
1/2	41 (17.2)	70 (31.3)		
3/4	197 (82.8)	154 (68.8)		
Primary pN stage			0.172	< 0.001*
0	110 (46.2)	142 (63.4)		
1/2	128 (53.8)	82 (36.6)		
Vascular invasion			0.061	0.189
No	204 (85.7)	201 (89.7)		
Yes	34 (14.3)	23 (10.3)		
Nerve invasion			0.054	0.246
No	215 (90.3)	209 (93.3)		
Yes	23 (9.7)	15 (6.7)		
Synchronous distant metastases			0.074	0.113
No	173 (72.7)	177 (79.0)		
Yes	65 (27.3)	47 (21.0)		
Postoperative adjuvant chemotherapy for patients without metastases			0.103	**0.055**
No	79 (45.7)	99 (55.9)		
Yes	94 (54.3)	78 (44.1)		
Treatment for metastases			0.04	0.964
Chemotherapy only	26 (40.0)	19 (40.4)		
Resection + chemotherapy	39 (60.0)	28 (59.6)		

CEA: carcino-embryonic antigen.

Correlation coefficient and *P* value: Cramer's V in Pearson χ^2^ test.

Bold font for *P* < 0.10;

* *P* < 0.05.

Synchronous distant metastases were defined as a diagnosis of distant metastases together with, or within a six-month interval of, diagnosis of the primary CRC. Pathological tumor stage was documented according to the AJCC TNM classification (version 7, 2010). For patients without synchronous distant metastases, postoperative adjuvant chemotherapy was given according to the Chinese and NCCN colorectal guidelines. For patients with resectable synchronous distant metastases, radical resections were conducted on the metastases. No anti-EGFR, anti-VEGF or other targeted agents were used.

### High HOXC6 expression in tumors indicated poor overall survival

**Figure 2:**
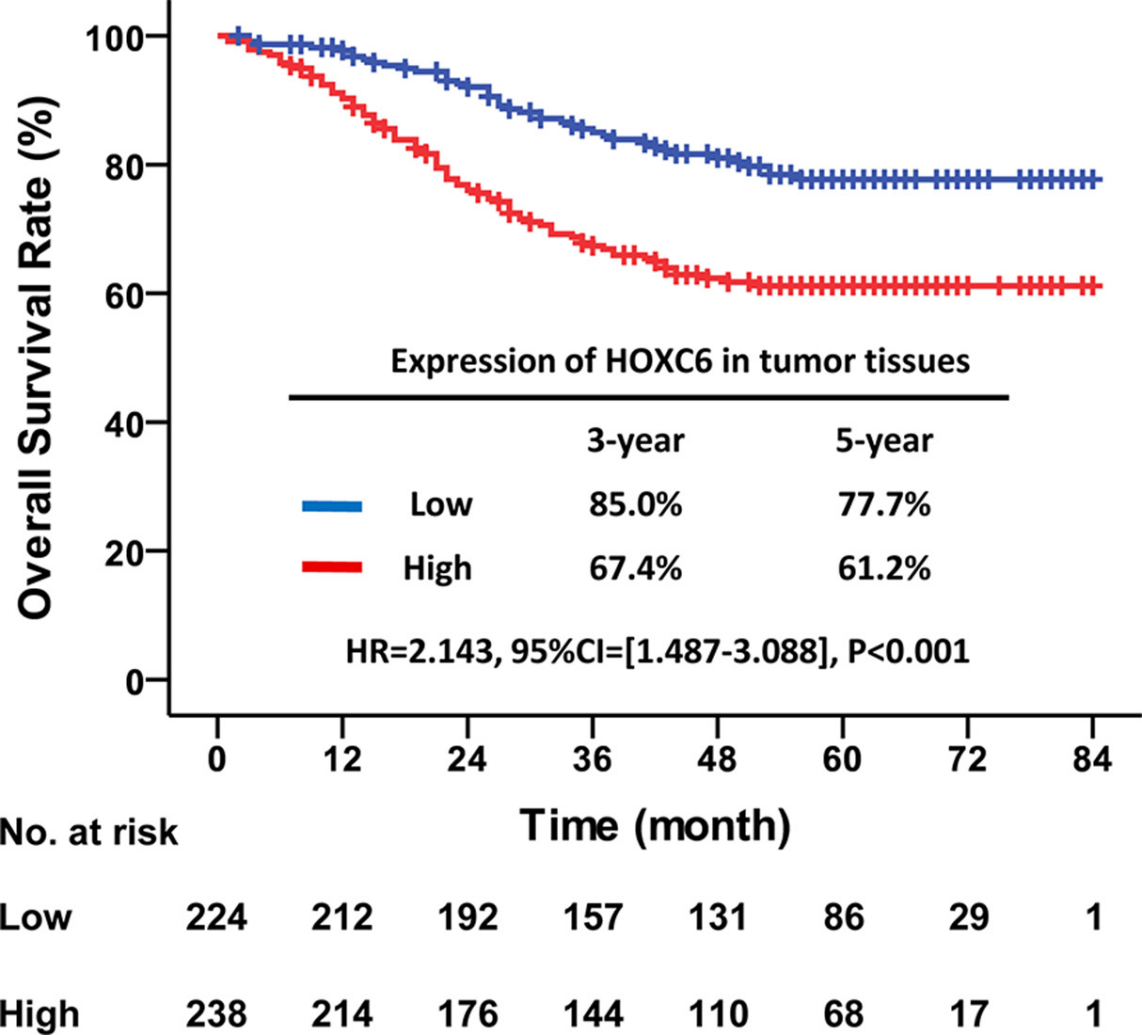
High HOXC6 expression in tumor tissues indicated poor OS Kaplan–Meier survival curve with HOXC6 expression in tumor tissues for CRC patients after primary tumor resection. HR: hazard ratio; CI: confidence interval; *P* value: log-rank test.

The median follow-up time for all patients was 51.0 months (IQR = [28.0–63.0]). OS of patients with high HOXC6 expression was significantly worse than that of patients with low expression (HR = 2.143, 95% CI = [1.487–3.088], *P* < 0.001). For patients with high HOXC6 expression, the 3- and 5-year OS rates were 67.4% and 61.2%, respectively. For patients with low HOXC6 expression, they were 85.0% and 77.7%, respectively (Figure 2). These results suggested that higher HOXC6 expression was associated with poorer OS.

In univariate analysis, OS was significantly associated with preoperative CEA (*P* < 0.001), primary tumor differentiation (*P* = 0.015), primary pT stage (*P* < 0.001), primary pN stage (*P* < 0.001), synchronous distant metastases (*P* < 0.001), HOXC6 expression (*P* < 0.001), postoperative adjuvant chemotherapy (*P* < 0.001) and treatment for metastases (P < 0.001) (Table 2). However, the multivariate analysis indicated that only primary pT stage (*P* = 0.032), primary pN stage (*P* = 0.024), synchronous distant metastases (*P* = 0.021), expression of HOXC6 (*P* = 0.028), postoperative adjuvant chemotherapy (*P* = 0.029) and treatment for metastases (*P* = 0.001) were independent prognostic factors. Thus, high HOXC6 expression was an independent risk factor for poor CRC patient prognosis.

**Table 2:** Univariate and multivariate analyses for OS

	Univariate analysis			Multivariate analysis		
	HR	95% CI	*P* value	HR	95% CI	*P* value
Sex						
Male	1	−	−	1	–	–
Female	1.045	0.736–1.485	0.804	1.110	0.765–1.611	0.581
Age - year						
≤ 60	1	–	–	1	–	–
> 60	1.136	0.805–1.603	0.467	1.026	0.713–1.477	0.889
Preoperative CEA - ng/ml						
< 5	1	–	–	1	–	–
≥ 5	2.444	1.699–3.515	< 0.001*	1.380	0.909–2.095	0.130
Preoperative neoadjuvant treatment						
No	1	–	–	1	–	–
Yes	1.336	0.933–1.914	0.114	0.790	0.526–1.187	0.257
Primary tumor site						
Right-sided	1	–	–	1	–	–
Left-sided	0.988	0.634–1.539	0.956	0.827	0.506–1.353	0.450
Rectum	0.836	0.552–1.266	0.398	0.715	0.451–1.133	0.153
Primary tumor size - cm						
≤ 4	1	–	–	1	–	–
> 4	1.113	0.786–1.575	0.546	1.178	0.805–1.725	0.399
Primary histological type						
Non-mucinous	1	–	–	1	–	–
Mucinous	1.257	0.788–2.006	0.338	1.002	0.599–1.675	0.994
Primary differentiation						
Well to moderate	1	–	–	1	–	–
Poor	1.562	1.090–2.238	0.015*	0.974	0.663–1.430	0.892
Primary pT stage						
1/2	1	–	–	1	–	–
3/4	3.680	2.031–6.665	< 0.001*	2.005	1.064–3.779	0.032*
Primary pN stage						
0	1	–	–	1	–	–
1/2	3.909	2.661–5.743	< 0.001*	1.753	1.077–2.853	0.024*
Vascular invasion						
No	1	–	–	1	–	–
Yes	1.343	0.757–2.383	0.313	1.354	0.722–2.539	0.345
Nerve invasion						
No	1	–	–	1	–	–
Yes	1.118	0.617–2.025	0.713	1.273	0.635–2.549	0.496
Synchronous distant metastases						
No	1	–	–	1	–	–
Yes	5.703	4.029–8.074	< 0.001*	4.810	1.269–18.231	0.021*
Expression of HOXC6 in tumor tissues						
Low	1	–	–	1	–	–
High	2.143	1.487–3.088	< 0.001*	1.563	1.049–2.328	0.028*
Postoperative adjuvant chemotherapy for patients without metastases						
No	1	–	–	1	–	–
Yes	3.388	1.918–5.985	< 0.001*	2.238	1.087–4.609	0.029*
Treatment for metastases						
Chemotherapy only	1	–	–	1	–	–
Resection + chemotherapy	0.125	0.085–0.184	< 0.001*	0.384	0.218–0.679	0.001*

HR: hazard ratio; CI: confidence interval; CEA: carcino-embryonic antigen.

Bold font for *P* < 0.10;

* *P* < 0.05.

Synchronous distant metastases were defined as a diagnosis of distant metastases together with, or within a six-month interval of, diagnosis of the primary CRC. Pathological tumor stage was documented according to the AJCC TNM classification (version 7, 2010). For patients without synchronous distant metastases, postoperative adjuvant chemotherapy was given according to the Chinese and NCCN colorectal guidelines. For patients with resectable synchronous distant metastases, radical resections were conducted on the metastases. No anti-EGFR, anti-VEGF or other targeted agents were used.

### HOXC6 downregulation inhibited tumor cell proliferation *in vitro* and *in vivo*

**Figure 3:**
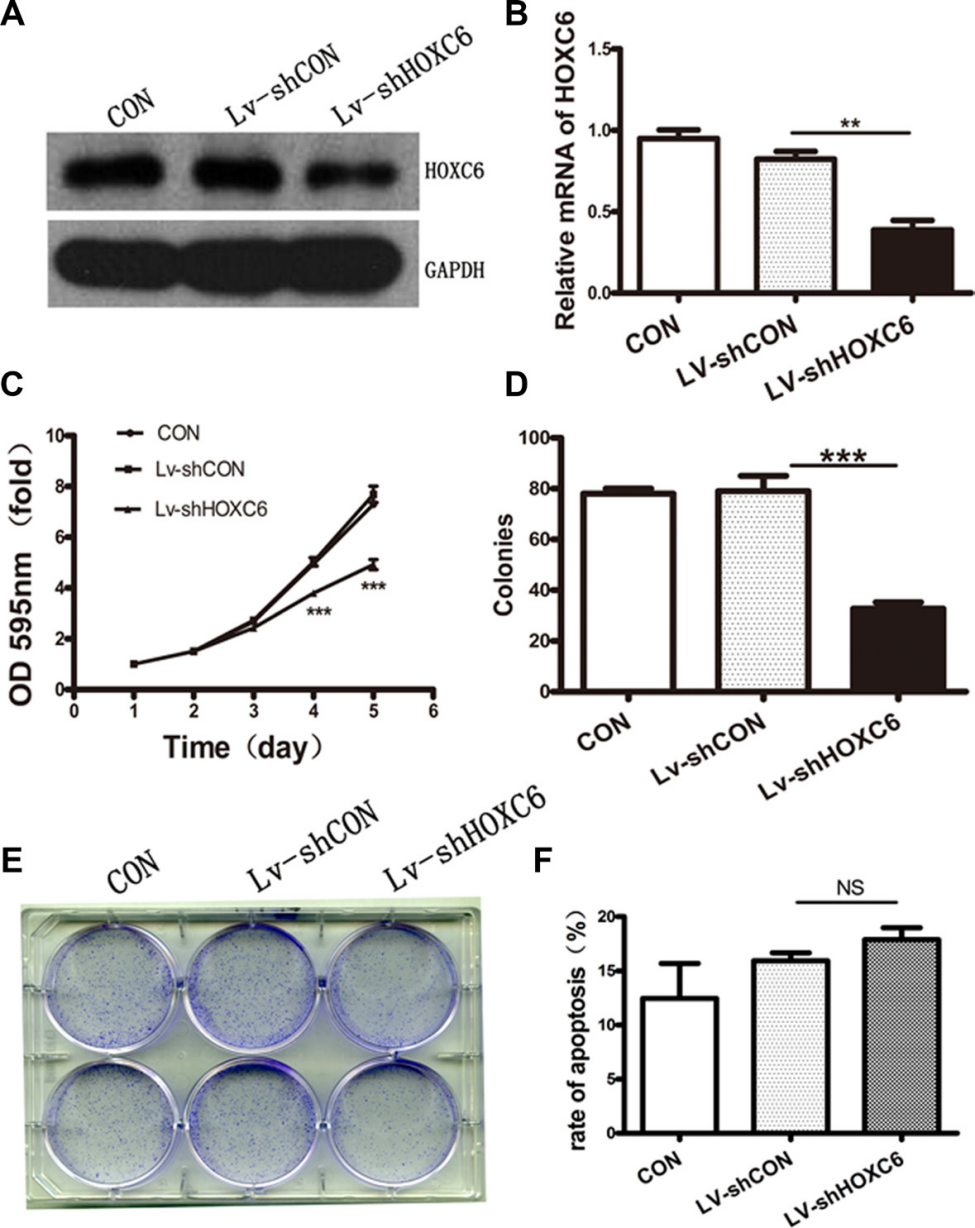
HOXC6 downregulation inhibited tumor cell proliferation *in vitro* Western blotting analysis for HOXC6 in untreated (CON) and Lv-shCON- or Lv-shHOXC6-infected HCT116 cells, with GAPDH as the control (**A**) RT-PCR analysis of HOXC6 mRNA levels with GAPDH as the control (**B**) CON, Lv-shCON and Lv-shHOXC6 group growth curves as measured by MTT assay (**C**) Colony counts (**D**) and images (**E**) for CON cells and Lv-shCON- or Lv-shHOXC6-infeced cells. Apoptosis was assayed by flow cytometry (**F**) **P < 0.01; ***p < 0.001.

HCT116 cells were infected with lentivirus containing either shRNA targeting HOXC6 (shHOXC6) or control scrambled shRNA (shCON). Western blotting and real-time PCR analysis showed that shHOXC6 effectively decreased both HOXC6 protein and mRNA levels (Figure 3A–3B). In the first five days following treatment, shHOXC6 significantly reduced cell viability, as measured by MTT assay (*P* < 0.001) (Figure 3C). Under the same conditions, HCT116 cell colony formation was significantly reduced as shown by Giemsa staining (*P* < 0.001) (Figure 3D–3E). Apoptosis rate, as assayed by Annexin V-PI and flow cytometry, was slightly reduced in shHOXC6-treated cells compared with controls, although differences were not significant (Figure 3F).

**Figure 4:**
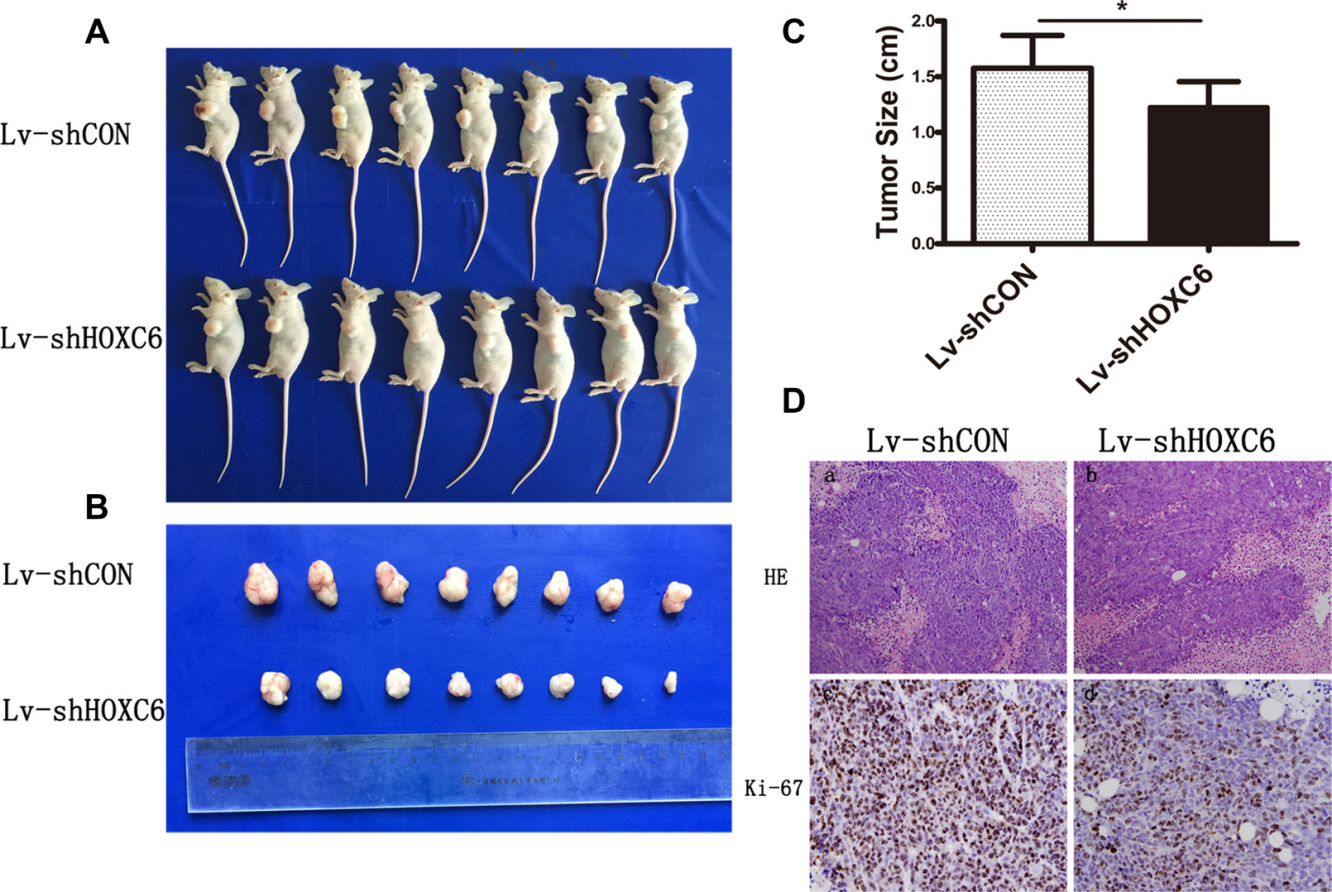
HOXC6 downregulation inhibited tumor growth *in vivo* Representative photos of mice (**A**) and tumors (**B**) were taken after sacrifice on day 18, and tumor size was measured (**C**) *P < 0.05 compared with control group. Ki-67 expression in Lv-shHOXC6-infected tumors was less than in controls as shown by H&E and IHC staining (**D**).

HCT116 cells stably expressing control or HOXC6 shRNA were injected subcutaneously into nude mice to form xenograft tumors. Eighteen days after injection, mice were sacrificed and tumor size was measured. HOXC6 downregulation reduced tumor growth rate (Figure 4A–4C). In addition, ki-67 expression, a marker of proliferation, was weaker in tumors infected with Lv-shHOXC6 as detected by IHC (Figure 4D). Taken together, these results suggest that HOXC6 may be a tumor growth-promoting oncogene.

### HOXC6 downregulation inhibited autophagy

Autophagy regulation is an evolutionarily conserved function of HOX proteins [22], and overlaps closely with signaling pathways that regulate tumorigenesis [18]. In *Drosophila*, HOX proteins impose temporality on developmental autophagy and act as environmental signal effectors in starvation-induced autophagy.

**Figure 5:**
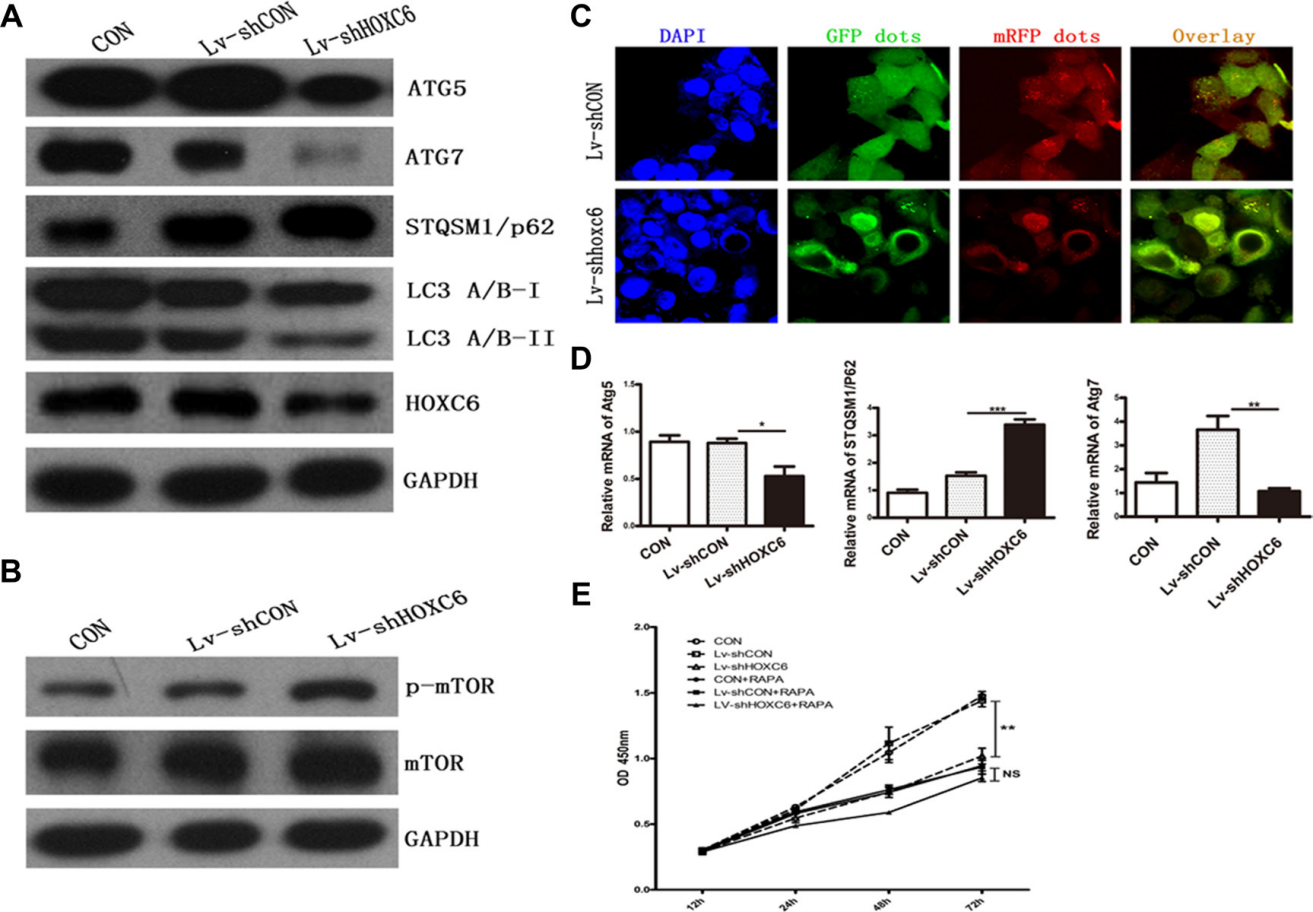
HOXC6 downregulation inhibited autophagy Following HCT116 cell infection with LV-shHOXC6 or LV-shCON, autophagy genes were analyzed by western blotting (**A**) mTOR pathway activity was detected by western blotting (**B**) Representative immunofluorescent images showing LC3 staining in cells infected with mRFP-GFP-LC3 adenovirus (nuclei are stained with DAPI) (**C**) Scale bar, 10 μm. Autophagy-related gene mRNA levels were detected by RT-PCR (**D**) The viability of HOXC6-downregulated cells treated with an autophagy activator was measured via CCK-8 assay (**E**).

Levels of the autophagy marker, LC3II/I, were decreased in shHOXC6-treated cells compared to controls, along with the autophagy-related molecules Atg5 and Atg7 (Figure 5A). A fluorescence assay using of a tandem monomeric mRFP- GFP-tagged LC3 is utilized to monitor flux, colocalization of both GFP and mRFP fluorescence indicates a compartment that has not fused with a lysosome, such as the phagophore or an autophagosome. In contrast, an mRFP signal without GPF corresponds to an autolysosome [23]. In our experiment, we found less red punta(mRFP signal only) in Lv-shHOXC6 than in control. The conversion process from autophagosme to autolysosome is suppressed which meant decreased autophagic flux (Figure 5C). In addition, defects in autophagy can cause accumulation of the autophagy receptor and substrate, STQSM1/P62. In our experiment, STQSM1/P62 was elevated. These findings agreed with mRNA levels measured by real-time PCR. Finally, the upstream mTOR pathway is a negative regulator of autophagy, and activation of mTOR suppresses autophagy [24]. We found significant upregulation of p-mTOR in cells infected with Lv-shHOXC6 (Figure 5B). Following treatment with rapamycin, an autophagy activator, reductions in cell proliferation were largely reversed. HOXC6 downregulation promoted mTOR activity, which in turn inhibited autophagy.

## DISCUSSION

HOXC6 is crucial to epithelial cell development and proliferation in response to hormonal signals. Moreover, HOXC6 deregulation has been detected in several cancer types. Although HOXC6 is reportedly differentially expressed in metastatic CRC [17], the exact role of HOXC6 in CRC initiation and progression remains unclear.

In this study, we found that HOXC6 expression is increased in CRC as compared to adjacent normal tissues. Clinicopathologic data showed that increased HOXC6 expression is correlated with tumor location in the right colon and higher T and N stages. Moreover, patients with higher HOXC6 expression had reduced OS. This indicated that abnormal HOXC6 expression might be associated with CRC development and progression.

HOXC6 promotes proliferation and cell survival by preventing apoptosis or promoting cell cycle progression. HOXC6 expression in cultured human BON1 carcinoid cells enhanced cell proliferation and activated the oncogenic activator protein-1 signaling pathway through JunD [14]. In head and neck squamous cell carcinoma, HOXC6 inhibits apoptosis via Bcl-2 regulation [15]. Moreover, HOXC6 was overexpressed in drug-resistant cells compared with parental cell lines and activated MDR-1 promoter activity, indicating chemotherapeutic drug resistance via MDR-1 regulation [16]. Although HOX genes are homeodomain transcription factors that recognize a consensus TAAT motif, their functions in cancer may be more diverse [4].

To explore the function of HOXC6 in CRC, we knocked down HOXC6 in HCT116 cells via shRNA. Both MTT and colony formation assays showed that HOXC6 downregulation decreased HCT116 cell proliferation. In addition, HOXC6 knockdown suppressed CRC cell growth in nude mouse xenografts.

Previous studies show that the PI3K/Akt pro-proliferative pathway is regulated by the direct HOXC6 targets BMP7, IGFBP3 and PDGFRA [25–28], suggesting that HOXC6 may enhance cell survival through PI3K/Akt signaling. HOXC6 may also promote cell survival through regulation of androgen receptor (AR) signaling and repressed expression of filamin A (FLNA), a cytoplasmic actin filament crosslinker [8]. We hypothesized that HOXC6 knockdown-mediated CRC cell proliferation inhibition may be regulated by the autophagy pathway. Western blotting and RT-PCR results showed that autophagy flux decreased along with expression of autophagy-associated genes, such as Atg5 and Atg7. In contrast, P62/SQSTM1 increased. Autophagy is responsible for p62 degradation; therefore, autophagy impairment is usually accompanied by p62 accumulation followed by the formation of p62- and ubiquitin-positive aggregate structures [29–31]. Moreover, the mTOR pathway, which negatively regulates autophagy, was activated as evidenced by elevated phosphorylated mTOR relative to total mTOR. These findings together suggest that HOXC6 may promote CRC progression via regulation of autophagy.

Autophagy is thought to play a dual role in cancer: it can prevent tumor initiation by suppressing chronic tissue damage, inflammation and genome instability via its quality control function or can sustain tumor metabolism, growth and survival via nutrient recycling. Current evidence indicates that these roles depend on cancer subtype, tumor stage and other factors [21, 32]. In our study, HOXC6 downregulation decreased CRC cell proliferation and tumorigenicity, and both of these effects correlated with autophagy pathway inhibition. HOXC6 may induce autophagy, which then promotes CRC progression. Whether HOXC6 directly or indirectly regulates autophagy should be further explored. Our results also suggested that HOXC6 could be a potential therapeutic marker. In conclusion, HOXC6 was abundantly expressed in CRC tissues compared to adjacent normal tissues from a large clinical cohort, and was negatively associated with OS. In addition, HOXC6 downregulation decreased CRC cell growth and this effect was mediated by the inhibition of autophagy.

## MATERIALS AND METHODS

### Cell culture

The human colorectal cancer cell line HCT116 was maintained in DMEM medium supplemented with 10% fetal bovine serum and 100 units/ml penicillin-streptomycin (Invitrogen, USA) at 37°C in 5% CO_2_.

### Patient samples

A human CRC tissue microarray (TMA) was constructed containing 462 paired tumor and normal mucosa tissues from patients recruited between June 2008 and October 2012 from Zhongshan Hospital of Fudan University (Shanghai, China). Clinicopathologic data were retrieved from our prospectively constructed CRC database. Prospective data collection and quality management were performed by an independent full-time research assistant. All patients received radical primary tumor resections. Tumor stage was determined according to the seventh edition of the International Union Against Cancer (UICC)/American Joint Committee on Cancer (AJCC) TNM classification. For patients without synchronous distant metastases, postoperative adjuvant chemotherapy was given according to the Chinese and NCCN colorectal guidelines. For patients with resectable synchronous distant metastases, radical resections were also conducted on the metastases. For patients with unresectable distant metastases, chemotherapy was given as treatment. No anti-EGFR, anti-VEGF or other targeted agents were used. Ethical approval was obtained from the Clinical Research Ethics Committee of Zhongshan Hospital of Fudan University. Signed informed consent was obtained from all patients for the acquisition and use of tissue samples and clinical data. OS was calculated from the day of surgery to the date of death due to CRC or last follow-up.

### Immunohistochemistry

HOXC6 expression was measured in tumor samples using a Histostain^®^-Plus 3rd Gen IHC Detection Kit (Invitrogen, USA #85–9073) following the manufacturer's instructions. The tissue microarray (TMA) slide was dried overnight at 37°C, deparaffinized in xylene, rehydrated through graded alcohol, immersed in 3% hydrogen peroxide for 20 min to block endogenous peroxidase activity, and then antigen-retrieved by microwave heating in 0.01 M sodium citrate buffer (pH = 6.0) at 100°C for 30 min. Subsequently, slides were pre-incubated with 10% normal goat serum at room temperature (RT) for 30 min to reduce nonspecific reaction. The primary rabbit anti-HOXC6 polyclonal antibody (ab41587, Abcam) was diluted (1:50) in phosphate-buffered saline (PBS) with 3% bovine serum albumin (BSA) and applied overnight in a humidity chamber at 4°C. Slides were incubated with a polymer peroxidase-labeled secondary antibody for 30 min at RT and then stained with DAB. Finally, sections were counterstained with hematoxylin.

HOXC6 expression was calculated as the product of two independent scores, the proportion of positive tumor cells in the tissues and the average intensity of positive tumor cells in the tumor tissues (no staining = 0, weak staining = 1, moderate staining = 2 and strong staining = 3). The final score was classified as low- or high-expression using the median value as the cut-off. Scores were determined independently by two senior pathologists.

For statistical analyses, categorical parameters were compared using a two-sided Pearson's χ2 test or Fisher's exact test, as appropriate. Correlation coefficients were calculated using Cramer's V. The normal continuous parameters were compared using Student's *t*-test and reported as means ± standard deviations (SD). The abnormal continuous parameters were compared using the Wilcoxon rank test and reported as medians with interquartile ranges (IQR). The summary statistics on time-to-event variables were calculated according to the Kaplan–Meier method and were compared using the log-rank test. The hazard ratio (HR) of survival was calculated using Cox regression and reported as a 95% confidence interval (CI). Univariate and multivariate analyses were also conducted using Cox regression. *P* < 0.05 was considered statistically significant.

### Lentivirus packaging

Short hairpin RNA (shRNA) targeting the human HOXC6 gene (NM_004503) was designed as follows: sense a 5ʹ-CCGGGACCTCAATCGCTCAGGATTT CTCGAG AA ATCCTGAGCGATTGAGGTCTTTTTG-3ʹ, sense b 5ʹ-AATTCAA A A AGACCTCAATCGCTCA GGATTTCTCGAGAAATCCTGAGCGATTGAGGTC-3ʹ.

Both shRNAs were inserted into the vector pFH-L (Shanghai Hollybio, China) between restriction enzyme sites for BamHI and EcoRI. The shRNA-harboring plasmid and two helper plasmids, pVSVG-I and pCMVΔR8.92 (Shanghai Hollybio, China), were transfected into HEK293T cells using Lipofectamine 2000 (Life Technologies, USA). Two days after transfection, cell culture media was collected and concentrated. The recombinant lentivirus was stored at −80°C.

### MTT assay

Lentivirus-infected HCT116 cells were seeded in 96-well plates at 2,000 cells/well. At 1, 2, 3, 4 and 5 days post incubation, MTT (3-(4, 5-dimethylthiazol-2-yl)-2, 5-diphenyltetrazolium bromide) solution was added to each well and incubated at 37°C for 4 h. Then, 100 μL acidic isopropanol (10% SDS, 5% isopropanol and 0.01 mol/L HCl) was added to each well after the medium was carefully removed. Plates were read 595 nm. Experiments were performed at least three times.

### Colony formation assay

HCT116 cells (200 cells/well) were seeded in 6-well plates 6 days after lentivirus infection. The medium was changed at 3-day intervals. After 8 days at 37°C with 5% CO2, cells were fixed with 4% paraformaldehyde and stained with freshly prepared diluted Giemsa stain for 20 min. Excess dye was washed off with double-distilled water.

### Quantitative real-time PCR

HCT116 cells were harvested six days after lentiviral infection. Total cellular RNA was extracted using TRIzol reagent (Life Technologies, USA) according to the manufacturer's instructions. RNA (2 μg) was reverse transcribed using a PrimeScript RT Reagent Kit (TaKaRa, Japan). Real-time PCR was performed using SYBR master mix (TaKaRa) on the Bio-Rad Connect Real-Time PCR platform. The primer sequences used for human HOXC6 were 5ʹ-CACCGCCTATGATCCAGTGAGG CA-3ʹ (forward) and 5ʹ-GCTGGAACTGAACACGACATTCTC-3ʹ (reverse). Relative mRNA quantities were determined using comparative cycle threshold methods and normalized against GAPDH.

### Western blotting

Lentivirus-infected HCT116 cells were collected, washed with ice-cold PBS and lysed in 2 × SDS sample buffer (100 mM Tris-HCl (pH 6.8), 10 mM EDTA, 4% SDS, 10% glycine). Samples containing equal amounts of protein (30 μg) were run on a 10% SDS-PAGE gel at 50 V for 3 h. Proteins were then transferred to a polyvinylidene fluoride (PVDF) membrane at 300 mA for 1.5 h. Membranes were blocked using 1% BSA in TBST at RT for 1 h and then incubated with the primary antibodies anti-HOXC6 (1:100, Novus, NB100-92309), autophagy markers LC3A/B, Atg5, and Atg7 (1: 1000, Cell Signaling Technology, Autophagy Antibody Sampler Kit #4445), anti-SQSTM1/p62 (1:500, Cell Signaling Technology, #8025) or anti-mTOR and p-mTOR (1:1000, Cell Signaling Technology, #9964S) overnight at 4°C. After washing in TBST, membranes were incubated with horseradish peroxidase-conjugated goat anti-mouse (1:5,000, Santa Cruz, SC-2005) and goat anti-rabbit (1:5,000, Santa Cruz, SC-2054) secondary antibodies for 1 h at RT. Membranes were washed in TBS-T and signals were detected using an enhanced chemiluminescence kit (Pierce, USA).

### *In vivo* tumorigenicity assay

All animal experiments were performed in accordance with NIH guidelines for the use of experimental animals. Male Nu/Nu mice, obtained from the Shanghai Laboratory Animal Center (SLAC), were injected left forelimb subcutaneously with 8 × 10^6^ stable monoclonal HCT116 cells infected with lentivirus harboring either shHOXC6 or a control shRNA to establish a CRC tumorigenicity model. After 18 days, eight mice were sacrificed, and subcutaneous tumors were collected for analysis. Tumor size was estimated from the maximum diameter (cm) of the tumor.

### Autophagy assay

Lentivirus-infected HCT116 were seeded on coverslips preprocessed in 40 μg/ml poly-lysine solution. mRFP-GFP-LC3 adenoviral vectors (HanBio, China) infection was performed according to the manufacturer's instructions. After 48 h, cells were fixed with 4% paraformaldehyde and stained by DAPI. Autophagy was observed under a fluorescence microscope (Olympus fv1200, Japan). Autophagic flux was determined by evaluating the number of GFP and mRFP puncta.

### CCK-8 assay

Lentivirus-infected HCT116 were seeded in 96-well plates at a density of 3000 cells per well, then exposed to rapamycin(1 μM) and its control reagents. After treatment, 10 ul of CCK-8 agent (Dojindo CK04, Japan) was added to each well and incubated at 37°C for another 2 h. The optical density of the solution was acquired at 450 nm with a miroplate spectrophotometer (Bio-Tek Epoch, USA).

### Statistical analysis

Comparisons between groups were performed using paired Student's *t* tests, and differences were considered statistically significant at *P* < 0.05. Data were presented as the mean ± standard deviation (SD) of three independent experiments.
